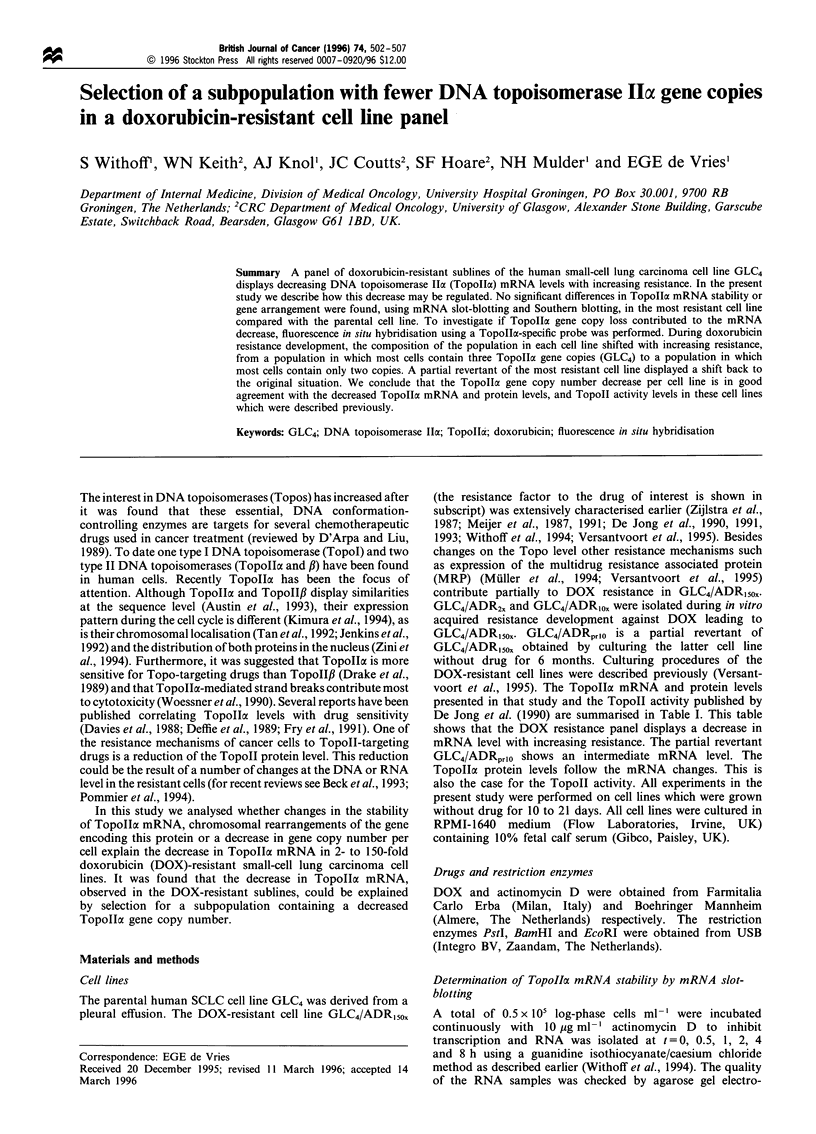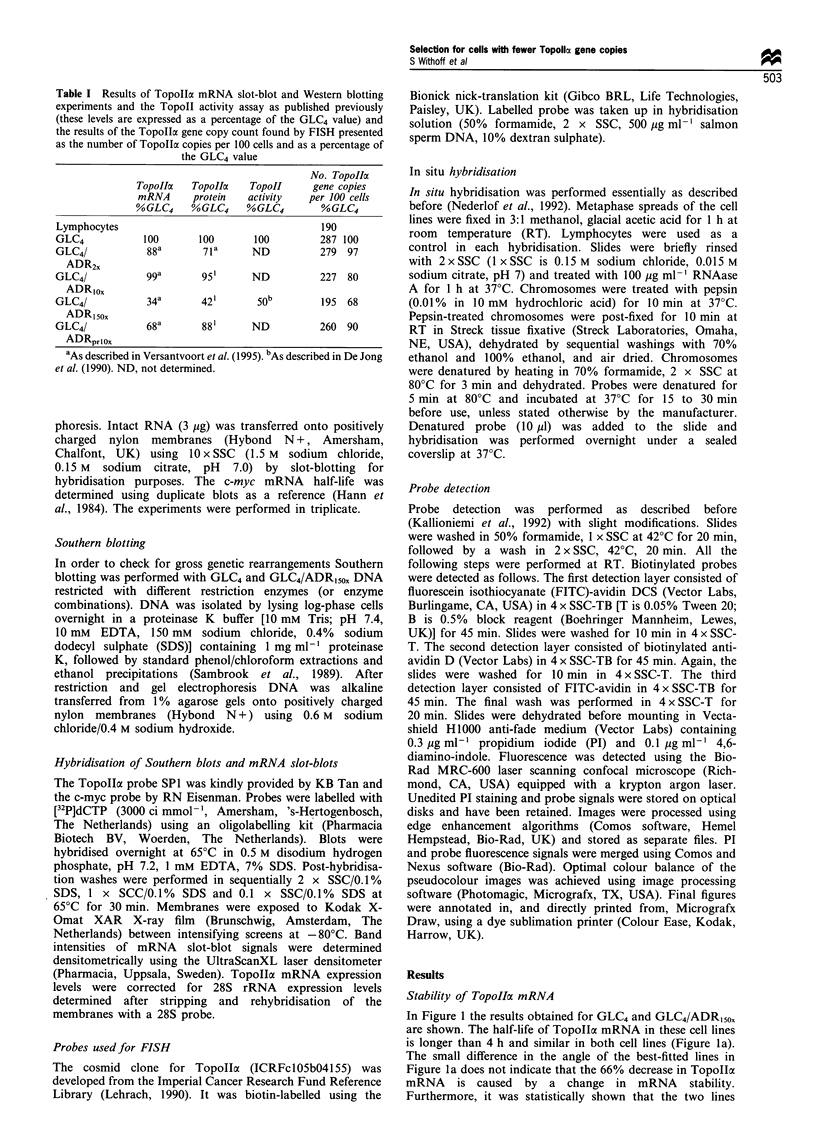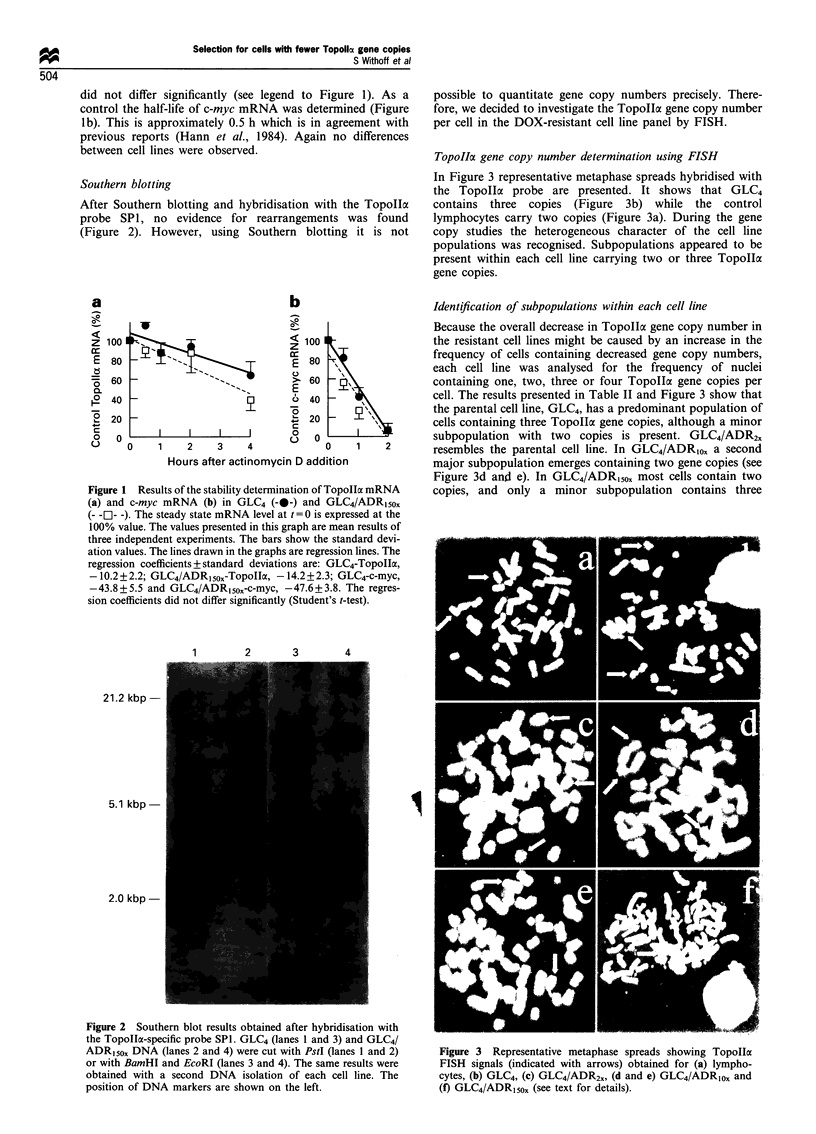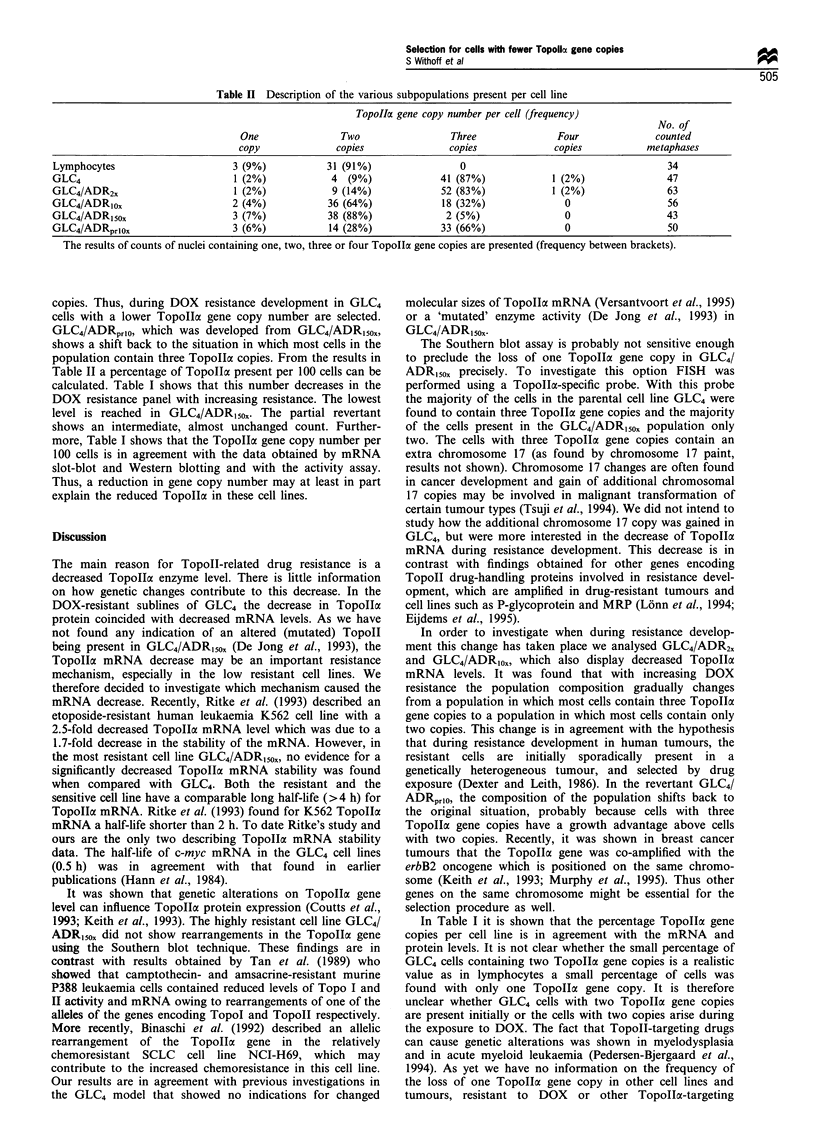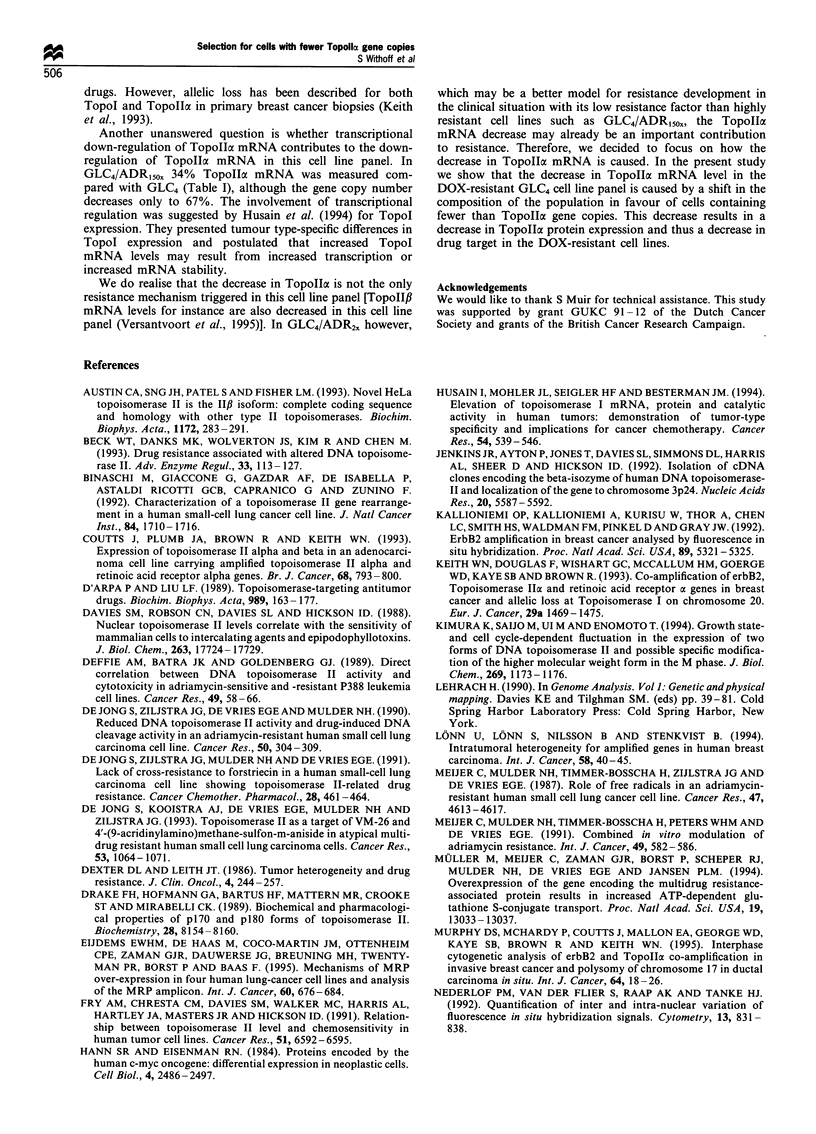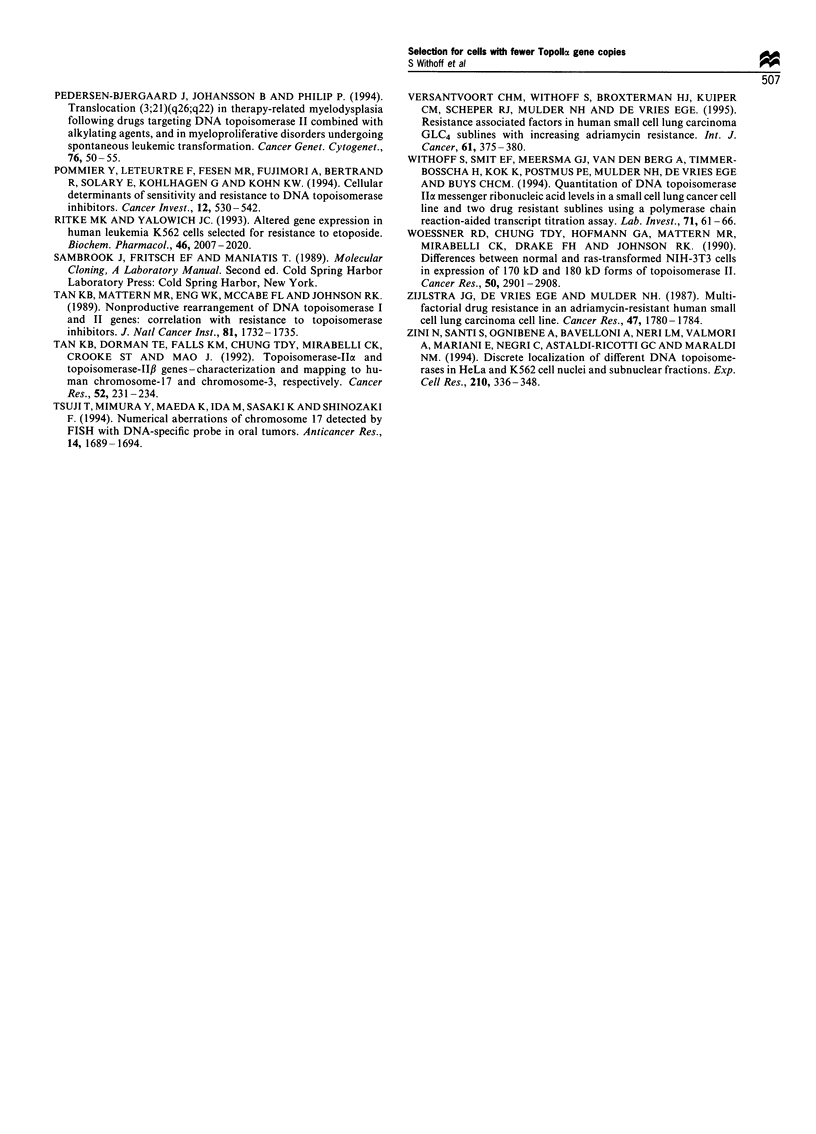# Selection of a subpopulation with fewer DNA topoisomerase II alpha gene copies in a doxorubicin-resistant cell line panel.

**DOI:** 10.1038/bjc.1996.393

**Published:** 1996-08

**Authors:** S. Withoff, W. N. Keith, A. J. Knol, J. C. Coutts, S. F. Hoare, N. H. Mulder, E. G. de Vries

**Affiliations:** Department of Internal Medicine, University Hospital Groningen, The Netherlands.

## Abstract

**Images:**


British Journal of Cancer (1996) 74, 502-507
© 1996 Stockton Press All rights reserved 0007-0920/96 $12.00

### Selection of a subpopulation with fewer DNA topoisomerase IIα gene copies in a doxorubicin-resistant cell line panel

S Withoff[1], WN Keith[2], AJ Knol[1], JC Coutts[2], SF Hoare[2], NH Mulder[1] and EGE de Vries[1]

*Department of Internal Medicine, Division of Medical Oncology, University Hospital Groningen, PO Box 30.001, 9700 RB Groningen, The Netherlands; [2]CRC Department of Medical Oncology, University of Glasgow, Alexander Stone Building, Garscube Estate, Switchback Road, Bearsden, Glasgow G61 1BD, UK.*

**Summary** A panel of doxorubicin-resistant sublines of the human small-cell lung carcinoma cell line $GLC_4$ displays decreasing DNA topoisomerase IIα (TopoIIα) mRNA levels with increasing resistance. In the present study we describe how this decrease may be regulated. No significant differences in TopoIIα mRNA stability or gene arrangement were found, using mRNA slot-blotting and Southern blotting, in the most resistant cell line compared with the parental cell line. To investigate if TopoIIα gene copy loss contributed to the mRNA decrease, fluorescence *in situ* hybridisation using a TopoIIα-specific probe was performed. During doxorubicin resistance development, the composition of the population in each cell line shifted with increasing resistance, from a population in which most cells contain three TopoIIα gene copies ($GLC_4$) to a population in which most cells contain only two copies. A partial revertant of the most resistant cell line displayed a shift back to the original situation. We conclude that the TopoIIα gene copy number decrease per cell line is in good agreement with the decreased TopoIIα mRNA and protein levels, and TopoII activity levels in these cell lines which were described previously.

**Keywords:** $GLC_4$; DNA topoisomerase IIα; TopoIIα; doxorubicin; fluorescence *in situ* hybridisation

The interest in DNA topoisomerases (Topos) has increased after it was found that these essential, DNA conformation-controlling enzymes are targets for several chemotherapeutic drugs used in cancer treatment (reviewed by D'Arpa and Liu, 1989). To date one type I DNA topoisomerase (TopoI) and two type II DNA topoisomerases (TopoIIα and β) have been found in human cells. Recently TopoIIα has been the focus of attention. Although TopoIIα and TopoIIβ display similarities at the sequence level (Austin *et al.*, 1993), their expression pattern during the cell cycle is different (Kimura *et al.*, 1994), as is their chromosomal localisation (Tan *et al.*, 1992; Jenkins *et al.*, 1992) and the distribution of both proteins in the nucleus (Zini *et al.*, 1994). Furthermore, it was suggested that TopoIIα is more sensitive for Topo-targeting drugs than TopoIIβ (Drake *et al.*, 1989) and that TopoIIα-mediated strand breaks contribute most to cytotoxicity (Woessner *et al.*, 1990). Several reports have been published correlating TopoIIα levels with drug sensitivity (Davies *et al.*, 1988; Deffie *et al.*, 1989; Fry *et al.*, 1991). One of the resistance mechanisms of cancer cells to TopoII-targeting drugs is a reduction of the TopoII protein level. This reduction could be the result of a number of changes at the DNA or RNA level in the resistant cells (for recent reviews see Beck *et al.*, 1993; Pommier *et al.*, 1994).

In this study we analysed whether changes in the stability of TopoIIα mRNA, chromosomal rearrangements of the gene encoding this protein or a decrease in gene copy number per cell explain the decrease in TopoIIα mRNA in 2- to 150-fold doxorubicin (DOX)-resistant small-cell lung carcinoma cell lines. It was found that the decrease in TopoIIα mRNA, observed in the DOX-resistant sublines, could be explained by selection for a subpopulation containing a decreased TopoIIα gene copy number.

### Materials and methods

### *Cell lines*

The parental human SCLC cell line $GLC_4$ was derived from a pleural effusion. The DOX-resistant cell line $GLC_4/ADR_{150x}$ (the resistance factor to the drug of interest is shown in subscript) was extensively characterised earlier (Zijlstra *et al.*, 1987; Meijer *et al.*, 1987, 1991; De Jong *et al.*, 1990, 1991, 1993; Withoff *et al.*, 1994; Versantvoort *et al.*, 1995). Besides changes on the Topo level other resistance mechanisms such as expression of the multidrug resistance associated protein (MRP) (Müller *et al.*, 1994; Versantvoort *et al.*, 1995) contribute partially to DOX resistance in $GLC_4/ADR_{150x}$. $GLC_4/ADR_{2x}$ and $GLC_4/ADR_{10x}$ were isolated during *in vitro* acquired resistance development against DOX leading to $GLC_4/ADR_{150x}$. $GLC_4/ADR_{pr10}$ is a partial revertant of $GLC_4/ADR_{150x}$ obtained by culturing the latter cell line without drug for 6 months. Culturing procedures of the DOX-resistant cell lines were described previously (Versantvoort *et al.*, 1995). The TopoIIα mRNA and protein levels presented in that study and the TopoII activity published by De Jong *et al.* (1990) are summarised in Table I. This table shows that the DOX resistance panel displays a decrease in mRNA level with increasing resistance. The partial revertant $GLC_4/ADR_{pr10}$ shows an intermediate mRNA level. The TopoIIα protein levels follow the mRNA changes. This is also the case for the TopoII activity. All experiments in the present study were performed on cell lines which were grown without drug for 10 to 21 days. All cell lines were cultured in RPMI-1640 medium (Flow Laboratories, Irvine, UK) containing 10% fetal calf serum (Gibco, Paisley, UK).

### *Drugs and restriction enzymes*

DOX and actinomycin D were obtained from Farmitalia Carlo Erba (Milan, Italy) and Boehringer Mannheim (Almere, The Netherlands) respectively. The restriction enzymes *Pst*I, *Bam*HI and *Eco*RI were obtained from USB (Integro BV, Zaandam, The Netherlands).

### *Determination of TopoIIα mRNA stability by mRNA slot-blotting*

A total of $0.5 \times 10^5$ log-phase cells $ml^{-1}$ were incubated continuously with 10 $\mu g\ ml^{-1}$ actinomycin D to inhibit transcription and RNA was isolated at $t = 0$, 0.5, 1, 2, 4 and 8 h using a guanidine isothiocyanate/caesium chloride method as described earlier (Withoff *et al.*, 1994). The quality of the RNA samples was checked by agarose gel electro-

Correspondence: EGE de Vries
Received 20 December 1995; revised 11 March 1996; accepted 14 March 1996

**Table I** Results of TopoIIα mRNA slot-blot and Western blotting experiments and the TopoII activity assay as published previously (these levels are expressed as a percentage of the $GLC_4$ value) and the results of the TopoIIα gene copy count found by FISH presented as the number of TopoIIα copies per 100 cells and as a percentage of the $GLC_4$ value

| | *TopoIIα mRNA %GLC₄* | *TopoIIα protein %GLC₄* | *TopoII activity %GLC₄* | *No. TopoIIα gene copies per 100 cells* | *%GLC₄* |
|---|---|---|---|---|---|
| Lymphocytes | | | | 190 | |
| $GLC_4$ | 100 | 100 | 100 | 287 | 100 |
| $GLC_4/ADR_{2x}$ | 88[a] | 71[a] | ND | 279 | 97 |
| $GLC_4/ADR_{10x}$ | 99[a] | 95[1] | ND | 227 | 80 |
| $GLC_4/ADR_{150x}$ | 34[a] | 42[1] | 50[b] | 195 | 68 |
| $GLC_4/ADR_{pr10x}$ | 68[a] | 88[1] | ND | 260 | 90 |

[a]As described in Versantvoort *et al.* (1995). [b]As described in De Jong *et al.* (1990). ND, not determined.

phoresis. Intact RNA (3 μg) was transferred onto positively charged nylon membranes (Hybond N+, Amersham, Chalfont, UK) using 10×SSC (1.5 M sodium chloride, 0.15 M sodium citrate, pH 7.0) by slot-blotting for hybridisation purposes. The c-*myc* mRNA half-life was determined using duplicate blots as a reference (Hann *et al.*, 1984). The experiments were performed in triplicate.

### *Southern blotting*

In order to check for gross genetic rearrangements Southern blotting was performed with $GLC_4$ and $GLC_4/ADR_{150x}$ DNA restricted with different restriction enzymes (or enzyme combinations). DNA was isolated by lysing log-phase cells overnight in a proteinase K buffer [10 mM Tris; pH 7.4, 10 mM EDTA, 150 mM sodium chloride, 0.4% sodium dodecyl sulphate (SDS)] containing 1 mg $ml^{-1}$ proteinase K, followed by standard phenol/chloroform extractions and ethanol precipitations (Sambrook *et al.*, 1989). After restriction and gel electrophoresis DNA was alkaline transferred from 1% agarose gels onto positively charged nylon membranes (Hybond N+) using 0.6 M sodium chloride/0.4 M sodium hydroxide.

### *Hybridisation of Southern blots and mRNA slot-blots*

The TopoIIα probe SP1 was kindly provided by KB Tan and the c-myc probe by RN Eisenman. Probes were labelled with [$^{32}$P]dCTP (3000 ci $mmol^{-1}$, Amersham, 's-Hertogenbosch, The Netherlands) using an oligolabelling kit (Pharmacia Biotech BV, Woerden, The Netherlands). Blots were hybridised overnight at 65°C in 0.5 M disodium hydrogen phosphate, pH 7.2, 1 mM EDTA, 7% SDS. Post-hybridisation washes were performed in sequentially 2 × SSC/0.1% SDS, 1 × SCC/0.1% SDS and 0.1 × SSC/0.1% SDS at 65°C for 30 min. Membranes were exposed to Kodak X-Omat XAR X-ray film (Brunschwig, Amsterdam, The Netherlands) between intensifying screens at −80°C. Band intensities of mRNA slot-blot signals were determined densitometrically using the UltraScanXL laser densitometer (Pharmacia, Uppsala, Sweden). TopoIIα mRNA expression levels were corrected for 28S rRNA expression levels determined after stripping and rehybridisation of the membranes with a 28S probe.

### *Probes used for FISH*

The cosmid clone for TopoIIα (ICRFc105b04155) was developed from the Imperial Cancer Research Fund Reference Library (Lehrach, 1990). It was biotin-labelled using the Bionick nick-translation kit (Gibco BRL, Life Technologies, Paisley, UK). Labelled probe was taken up in hybridisation solution (50% formamide, 2 × SSC, 500 μg $ml^{-1}$ salmon sperm DNA, 10% dextran sulphate).

### In situ *hybridisation*

*In situ* hybridisation was performed essentially as described before (Nederlof *et al.*, 1992). Metaphase spreads of the cell lines were fixed in 3:1 methanol, glacial acetic acid for 1 h at room temperature (RT). Lymphocytes were used as a control in each hybridisation. Slides were briefly rinsed with 2×SSC (1×SSC is 0.15 M sodium chloride, 0.015 M sodium citrate, pH 7) and treated with 100 μg $ml^{-1}$ RNAase A for 1 h at 37°C. Chromosomes were treated with pepsin (0.01% in 10 mM hydrochloric acid) for 10 min at 37°C. Pepsin-treated chromosomes were post-fixed for 10 min at RT in Streck tissue fixative (Streck Laboratories, Omaha, NE, USA), dehydrated by sequential washings with 70% ethanol and 100% ethanol, and air dried. Chromosomes were denatured by heating in 70% formamide, 2 × SSC at 80°C for 3 min and dehydrated. Probes were denatured for 5 min at 80°C and incubated at 37°C for 15 to 30 min before use, unless stated otherwise by the manufacturer. Denatured probe (10 μl) was added to the slide and hybridisation was performed overnight under a sealed coverslip at 37°C.

### *Probe detection*

Probe detection was performed as described before (Kallioniemi *et al.*, 1992) with slight modifications. Slides were washed in 50% formamide, 1×SSC at 42°C for 20 min, followed by a wash in 2×SSC, 42°C, 20 min. All the following steps were performed at RT. Biotinylated probes were detected as follows. The first detection layer consisted of fluorescein isothiocyanate (FITC)-avidin DCS (Vector Labs, Burlingame, CA, USA) in 4×SSC-TB [T is 0.05% Tween 20; B is 0.5% block reagent (Boehringer Mannheim, Lewes, UK)] for 45 min. Slides were washed for 10 min in 4×SSC-T. The second detection layer consisted of biotinylated anti-avidin D (Vector Labs) in 4×SSC-TB for 45 min. Again, the slides were washed for 10 min in 4×SSC-T. The third detection layer consisted of FITC-avidin in 4×SSC-TB for 45 min. The final wash was performed in 4×SSC-T for 20 min. Slides were dehydrated before mounting in Vectashield H1000 anti-fade medium (Vector Labs) containing 0.3 μg $ml^{-1}$ propidium iodide (PI) and 0.1 μg $ml^{-1}$ 4,6-diamino-indole. Fluorescence was detected using the Bio-Rad MRC-600 laser scanning confocal microscope (Richmond, CA, USA) equipped with a krypton argon laser. Unedited PI staining and probe signals were stored on optical disks and have been retained. Images were processed using edge enhancement algorithms (Comos software, Hemel Hempstead, Bio-Rad, UK) and stored as separate files. PI and probe fluorescence signals were merged using Comos and Nexus software (Bio-Rad). Optimal colour balance of the pseudocolour images was achieved using image processing software (Photomagic, Micrografx, TX, USA). Final figures were annotated in, and directly printed from, Micrografx Draw, using a dye sublimation printer (Colour Ease, Kodak, Harrow, UK).

### Results

### *Stability of TopoIIα mRNA*

In Figure 1 the results obtained for $GLC_4$ and $GLC_4/ADR_{150x}$ are shown. The half-life of TopoIIα mRNA in these cell lines is longer than 4 h and similar in both cell lines (Figure 1a). The small difference in the angle of the best-fitted lines in Figure 1a does not indicate that the 66% decrease in TopoIIα mRNA is caused by a change in mRNA stability. Furthermore, it was statistically shown that the two lines did not differ significantly (see legend to Figure 1). As a control the half-life of c-*myc* mRNA was determined (Figure 1b). This is approximately 0.5 h which is in agreement with previous reports (Hann *et al.*, 1984). Again no differences between cell lines were observed.

### *Southern blotting*

After Southern blotting and hybridisation with the TopoIIα probe SP1, no evidence for rearrangements was found (Figure 2). However, using Southern blotting it is not possible to quantitate gene copy numbers precisely. Therefore, we decided to investigate the TopoIIα gene copy number per cell in the DOX-resistant cell line panel by FISH.

### *TopoIIα gene copy number determination using FISH*

In Figure 3 representative metaphase spreads hybridised with the TopoIIα probe are presented. It shows that $GLC_4$ contains three copies (Figure 3b) while the control lymphocytes carry two copies (Figure 3a). During the gene copy studies the heterogeneous character of the cell line populations was recognised. Subpopulations appeared to be present within each cell line carrying two or three TopoIIα gene copies.

### *Identification of subpopulations within each cell line*

Because the overall decrease in TopoIIα gene copy number in the resistant cell lines might be caused by an increase in the frequency of cells containing decreased gene copy numbers, each cell line was analysed for the frequency of nuclei containing one, two, three or four TopoIIα gene copies per cell. The results presented in Table II and Figure 3 show that the parental cell line, $GLC_4$, has a predominant population of cells containing three TopoIIα gene copies, although a minor subpopulation with two copies is present. $GLC_4/ADR_{2x}$ resembles the parental cell line. In $GLC_4/ADR_{10x}$ a second major subpopulation emerges containing two gene copies (see Figure 3d and e). In $GLC_4/ADR_{150x}$ most cells contain two copies, and only a minor subpopulation contains three

**Figure 1** Results of the stability determination of TopoIIα mRNA (**a**) and c-*myc* mRNA (**b**) in $GLC_4$ (-●-) and $GLC_4/ADR_{150x}$ (- -□- -). The steady state mRNA level at $t=0$ is expressed at the 100% value. The values presented in this graph are mean results of three independent experiments. The bars show the standard deviation values. The lines drawn in the graphs are regression lines. The regression coefficients ± standard deviations are: $GLC_4$-TopoIIα, $-10.2 \pm 2.2$; $GLC_4/ADR_{150x}$-TopoIIα, $-14.2 \pm 2.3$; $GLC_4$-c-myc, $-43.8 \pm 5.5$ and $GLC_4/ADR_{150x}$-c-myc, $-47.6 \pm 3.8$. The regression coefficients did not differ significantly (Student's *t*-test).

**Figure 2** Southern blot results obtained after hybridisation with the TopoIIα-specific probe SP1. $GLC_4$ (lanes 1 and 3) and $GLC_4/ADR_{150x}$ DNA (lanes 2 and 4) were cut with *Pst*I (lanes 1 and 2) or with *Bam*HI and *Eco*RI (lanes 3 and 4). The same results were obtained with a second DNA isolation of each cell line. The position of DNA markers are shown on the left.

**Figure 3** Representative metaphase spreads showing TopoIIα FISH signals (indicated with arrows) obtained for (**a**) lymphocytes, (**b**) $GLC_4$, (**c**) $GLC_4/ADR_{2x}$, (**d** and **e**) $GLC_4/ADR_{10x}$ and (**f**) $GLC_4/ADR_{150x}$ (see text for details).

**Table II** Description of the various subpopulations present per cell line

| | *TopoIIα gene copy number per cell (frequency)* | | | | |
|---|---|---|---|---|---|
| | *One copy* | *Two copies* | *Three copies* | *Four copies* | *No. of counted metaphases* |
| Lymphocytes | 3 (9%) | 31 (91%) | 0 | | 34 |
| $GLC_4$ | 1 (2%) | 4 (9%) | 41 (87%) | 1 (2%) | 47 |
| $GLC_4/ADR_{2x}$ | 1 (2%) | 9 (14%) | 52 (83%) | 1 (2%) | 63 |
| $GLC_4/ADR_{10x}$ | 2 (4%) | 36 (64%) | 18 (32%) | 0 | 56 |
| $GLC_4/ADR_{150x}$ | 3 (7%) | 38 (88%) | 2 (5%) | 0 | 43 |
| $GLC_4/ADR_{pr10x}$ | 3 (6%) | 14 (28%) | 33 (66%) | 0 | 50 |

The results of counts of nuclei containing one, two, three or four TopoIIα gene copies are presented (frequency between brackets).

copies. Thus, during DOX resistance development in $GLC_4$ cells with a lower TopoIIα gene copy number are selected. $GLC_4/ADR_{pr10}$, which was developed from $GLC_4/ADR_{150x}$, shows a shift back to the situation in which most cells in the population contain three TopoIIα copies. From the results in Table II a percentage of TopoIIα present per 100 cells can be calculated. Table I shows that this number decreases in the DOX resistance panel with increasing resistance. The lowest level is reached in $GLC_4/ADR_{150x}$. The partial revertant shows an intermediate, almost unchanged count. Furthermore, Table I shows that the TopoIIα gene copy number per 100 cells is in agreement with the data obtained by mRNA slot-blot and Western blotting and with the activity assay. Thus, a reduction in gene copy number may at least in part explain the reduced TopoIIα in these cell lines.

### Discussion

The main reason for TopoII-related drug resistance is a decreased TopoIIα enzyme level. There is little information on how genetic changes contribute to this decrease. In the DOX-resistant sublines of $GLC_4$ the decrease in TopoIIα protein coincided with decreased mRNA levels. As we have not found any indication of an altered (mutated) TopoII being present in $GLC_4/ADR_{150x}$ (De Jong *et al*., 1993), the TopoIIα mRNA decrease may be an important resistance mechanism, especially in the low resistant cell lines. We therefore decided to investigate which mechanism caused the mRNA decrease. Recently, Ritke *et al.* (1993) described an etoposide-resistant human leukaemia K562 cell line with a 2.5-fold decreased TopoIIα mRNA level which was due to a 1.7-fold decrease in the stability of the mRNA. However, in the most resistant cell line $GLC_4/ADR_{150x}$, no evidence for a significantly decreased TopoIIα mRNA stability was found when compared with $GLC_4$. Both the resistant and the sensitive cell line have a comparable long half-life (>4 h) for TopoIIα mRNA. Ritke *et al.* (1993) found for K562 TopoIIα mRNA a half-life shorter than 2 h. To date Ritke's study and ours are the only two describing TopoIIα mRNA stability data. The half-life of c-*myc* mRNA in the $GLC_4$ cell lines (0.5 h) was in agreement with that found in earlier publications (Hann *et al*., 1984).

It was shown that genetic alterations on TopoIIα gene level can influence TopoIIα protein expression (Coutts *et al*., 1993; Keith *et al*., 1993). The highly resistant cell line $GLC_4/ADR_{150x}$ did not show rearrangements in the TopoIIα gene using the Southern blot technique. These findings are in contrast with results obtained by Tan *et al.* (1989) who showed that camptothecin- and amsacrine-resistant murine P388 leukaemia cells contained reduced levels of Topo I and II activity and mRNA owing to rearrangements of one of the alleles of the genes encoding TopoI and TopoII respectively. More recently, Binaschi *et al.* (1992) described an allelic rearrangement of the TopoIIα gene in the relatively chemoresistant SCLC cell line NCI-H69, which may contribute to the increased chemoresistance in this cell line. Our results are in agreement with previous investigations in the $GLC_4$ model that showed no indications for changed molecular sizes of TopoIIα mRNA (Versantvoort *et al*., 1995) or a 'mutated' enzyme activity (De Jong *et al*., 1993) in $GLC_4/ADR_{150x}$.

The Southern blot assay is probably not sensitive enough to preclude the loss of one TopoIIα gene copy in $GLC_4/ADR_{150x}$ precisely. To investigate this option FISH was performed using a TopoIIα-specific probe. With this probe the majority of the cells in the parental cell line $GLC_4$ were found to contain three TopoIIα gene copies and the majority of the cells present in the $GLC_4/ADR_{150x}$ population only two. The cells with three TopoIIα gene copies contain an extra chromosome 17 (as found by chromosome 17 paint, results not shown). Chromosome 17 changes are often found in cancer development and gain of additional chromosomal 17 copies may be involved in malignant transformation of certain tumour types (Tsuji *et al*., 1994). We did not intend to study how the additional chromosome 17 copy was gained in $GLC_4$, but were more interested in the decrease of TopoIIα mRNA during resistance development. This decrease is in contrast with findings obtained for other genes encoding TopoII drug-handling proteins involved in resistance development, which are amplified in drug-resistant tumours and cell lines such as P-glycoprotein and MRP (Lönn *et al*., 1994; Eijdems *et al*., 1995).

In order to investigate when during resistance development this change has taken place we analysed $GLC_4/ADR_{2x}$ and $GLC_4/ADR_{10x}$, which also display decreased TopoIIα mRNA levels. It was found that with increasing DOX resistance the population composition gradually changes from a population in which most cells contain three TopoIIα gene copies to a population in which most cells contain only two copies. This change is in agreement with the hypothesis that during resistance development in human tumours, the resistant cells are initially sporadically present in a genetically heterogeneous tumour, and selected by drug exposure (Dexter and Leith, 1986). In the revertant $GLC_4/ADR_{pr10}$, the composition of the population shifts back to the original situation, probably because cells with three TopoIIα gene copies have a growth advantage above cells with two copies. Recently, it was shown in breast cancer tumours that the TopoIIα gene was co-amplified with the *erb*B2 oncogene which is positioned on the same chromosome (Keith *et al*., 1993; Murphy *et al*., 1995). Thus other genes on the same chromosome might be essential for the selection procedure as well.

In Table I it is shown that the percentage TopoIIα gene copies per cell line is in agreement with the mRNA and protein levels. It is not clear whether the small percentage of $GLC_4$ cells containing two TopoIIα gene copies is a realistic value as in lymphocytes a small percentage of cells was found with only one TopoIIα gene copy. It is therefore unclear whether $GLC_4$ cells with two TopoIIα gene copies are present initially or the cells with two copies arise during the exposure to DOX. The fact that TopoII-targeting drugs can cause genetic alterations was shown in myelodysplasia and in acute myeloid leukaemia (Pedersen-Bjergaard *et al*., 1994). As yet we have no information on the frequency of the loss of one TopoIIα gene copy in other cell lines and tumours, resistant to DOX or other TopoIIα-targeting drugs. However, allelic loss has been described for both TopoI and TopoIIα in primary breast cancer biopsies (Keith et al., 1993).

Another unanswered question is whether transcriptional down-regulation of TopoIIα mRNA contributes to the down-regulation of TopoIIα mRNA in this cell line panel. In $GLC_4/ADR_{150x}$ 34% TopoIIα mRNA was measured compared with $GLC_4$ (Table I), although the gene copy number decreases only to 67%. The involvement of transcriptional regulation was suggested by Husain et al. (1994) for TopoI expression. They presented tumour type-specific differences in TopoI expression and postulated that increased TopoI mRNA levels may result from increased transcription or increased mRNA stability.

We do realise that the decrease in TopoIIα is not the only resistance mechanism triggered in this cell line panel [TopoIIβ mRNA levels for instance are also decreased in this cell line panel (Versantvoort et al., 1995)]. In $GLC_4/ADR_{2x}$ however, which may be a better model for resistance development in the clinical situation with its low resistance factor than highly resistant cell lines such as $GLC_4/ADR_{150x}$, the TopoIIα mRNA decrease may already be an important contribution to resistance. Therefore, we decided to focus on how the decrease in TopoIIα mRNA is caused. In the present study we show that the decrease in TopoIIα mRNA level in the DOX-resistant $GLC_4$ cell line panel is caused by a shift in the composition of the population in favour of cells containing fewer than TopoIIα gene copies. This decrease results in a decrease in TopoIIα protein expression and thus a decrease in drug target in the DOX-resistant cell lines.

**Acknowledgements**
We would like to thank S Muir for technical assistance. This study was supported by grant GUKC 91-12 of the Dutch Cancer Society and grants of the British Cancer Research Campaign.

### References

AUSTIN CA, SNG JH, PATEL S AND FISHER LM. (1993). Novel HeLa topoisomerase II is the IIβ isoform: complete coding sequence and homology with other type II topoisomerases. *Biochim. Biophys. Acta.*, **1172**, 283-291.

BECK WT, DANKS MK, WOLVERTON JS, KIM R AND CHEN M. (1993). Drug resistance associated with altered DNA topoisomerase II. *Adv. Enzyme Regul.*, **33**, 113-127.

BINASCHI M, GIACCONE G, GAZDAR AF, DE ISABELLA P, ASTALDI RICOTTI GCB, CAPRANICO G AND ZUNINO F. (1992). Characterization of a topoisomerase II gene rearrangement in a human small-cell lung cancer cell line. *J. Natl Cancer Inst.*, **84**, 1710-1716.

COUTTS J, PLUMB JA, BROWN R AND KEITH WN. (1993). Expression of topoisomerase II alpha and beta in an adenocarcinoma cell line carrying amplified topoisomerase II alpha and retinoic acid receptor alpha genes. *Br. J. Cancer*, **68**, 793-800.

D'ARPA P AND LIU LF. (1989). Topoisomerase-targeting antitumor drugs. *Biochim. Biophys. Acta*, **989**, 163-177.

DAVIES SM, ROBSON CN, DAVIES SL AND HICKSON ID. (1988). Nuclear topoisomerase II levels correlate with the sensitivity of mammalian cells to intercalating agents and epipodophyllotoxins. *J. Biol. Chem.*, **263**, 17724-17729.

DEFFIE AM, BATRA JK AND GOLDENBERG GJ. (1989). Direct correlation between DNA topoisomerase II activity and cytotoxicity in adriamycin-sensitive and -resistant P388 leukemia cell lines. *Cancer Res.*, **49**, 58-66.

DE JONG S, ZIJLSTRA JG, DE VRIES EGE AND MULDER NH. (1990). Reduced DNA topoisomerase II activity and drug-induced DNA cleavage activity in an adriamycin-resistant human small cell lung carcinoma cell line. *Cancer Res.*, **50**, 304-309.

DE JONG S, ZIJLSTRA JG, MULDER NH AND DE VRIES EGE. (1991). Lack of cross-resistance to forstriecin in a human small-cell lung carcinoma cell line showing topoisomerase II-related drug resistance. *Cancer Chemother. Pharmacol.*, **28**, 461-464.

DE JONG S, KOOISTRA AJ, DE VRIES EGE, MULDER NH AND ZIJLSTRA JG. (1993). Topoisomerase II as a target of VM-26 and 4'-(9-acridinylamino)methane-sulfon-m-aniside in atypical multidrug resistant human small cell lung carcinoma cells. *Cancer Res.*, **53**, 1064-1071.

DEXTER DL AND LEITH JT. (1986). Tumor heterogeneity and drug resistance. *J. Clin. Oncol.*, **4**, 244-257.

DRAKE FH, HOFMANN GA, BARTUS HF, MATTERN MR, CROOKE ST AND MIRABELLI CK. (1989). Biochemical and pharmacological properties of p170 and p180 forms of topoisomerase II. *Biochemistry*, **28**, 8154-8160.

EIJDEMS EWHM, DE HAAS M, COCO-MARTIN JM, OTTENHEIM CPE, ZAMAN GJR, DAUWERSE JG, BREUNING MH, TWENTYMAN PR, BORST P AND BAAS F. (1995). Mechanisms of MRP over-expression in four human lung-cancer cell lines and analysis of the MRP amplicon. *Int. J. Cancer*, **60**, 676-684.

FRY AM, CHRESTA CM, DAVIES SM, WALKER MC, HARRIS AL, HARTLEY JA, MASTERS JR AND HICKSON ID. (1991). Relationship between topoisomerase II level and chemosensitivity in human tumor cell lines. *Cancer Res.*, **51**, 6592-6595.

HANN SR AND EISENMAN RN. (1984). Proteins encoded by the human c-myc oncogene: differential expression in neoplastic cells. *Cell Biol.*, **4**, 2486-2497.

HUSAIN I, MOHLER JL, SEIGLER HF AND BESTERMAN JM. (1994). Elevation of topoisomerase I mRNA, protein and catalytic activity in human tumors: demonstration of tumor-type specificity and implications for cancer chemotherapy. *Cancer Res.*, **54**, 539-546.

JENKINS JR, AYTON P, JONES T, DAVIES SL, SIMMONS DL, HARRIS AL, SHEER D AND HICKSON ID. (1992). Isolation of cDNA clones encoding the beta-isozyme of human DNA topoisomerase-II and localization of the gene to chromosome 3p24. *Nucleic Acids Res.*, **20**, 5587-5592.

KALLIONIEMI OP, KALLIONIEMI A, KURISU W, THOR A, CHEN LC, SMITH HS, WALDMAN FM, PINKEL D AND GRAY JW. (1992). ErbB2 amplification in breast cancer analysed by fluorescence in situ hybridization. *Proc. Natl Acad. Sci. USA*, **89**, 5321-5325.

KEITH WN, DOUGLAS F, WISHART GC, MCCALLUM HM, GOERGE WD, KAYE SB AND BROWN R. (1993). Co-amplification of erbB2, Topoisomerase IIα and retinoic acid receptor α genes in breast cancer and allelic loss at Topoisomerase I on chromosome 20. *Eur. J. Cancer*, **29a** 1469-1475.

KIMURA K, SAIJO M, UI M AND ENOMOTO T. (1994). Growth state- and cell cycle-dependent fluctuation in the expression of two forms of DNA topoisomerase II and possible specific modification of the higher molecular weight form in the M phase. *J. Biol. Chem.*, **269**, 1173-1176.

LEHRACH H. (1990). In *Genome Analysis. Vol 1: Genetic and physical mapping*. Davies KE and Tilghman SM. (eds) pp. 39-81. Cold Spring Harbor Laboratory Press: Cold Spring Harbor, New York.

LÖNN U, LÖNN S, NILSSON B AND STENKVIST B. (1994). Intratumoral heterogeneity for amplified genes in human breast carcinoma. *Int. J. Cancer*, **58**, 40-45.

MEIJER C, MULDER NH, TIMMER-BOSSCHA H, ZIJLSTRA JG AND DE VRIES EGE. (1987). Role of free radicals in an adriamycin-resistant human small cell lung cancer cell line. *Cancer Res.*, **47**, 4613-4617.

MEIJER C, MULDER NH, TIMMER-BOSSCHA H, PETERS WHM AND DE VRIES EGE. (1991). Combined *in vitro* modulation of adriamycin resistance. *Int. J. Cancer*, **49**, 582-586.

MÜLLER M, MEIJER C, ZAMAN GJR, BORST P, SCHEPER RJ, MULDER NH, DE VRIES EGE AND JANSEN PLM. (1994). Overexpression of the gene encoding the multidrug resistance-associated protein results in increased ATP-dependent glutathione S-conjugate transport. *Proc. Natl Acad. Sci. USA*, **19**, 13033-13037.

MURPHY DS, MCHARDY P, COUTTS J, MALLON EA, GEORGE WD, KAYE SB, BROWN R AND KEITH WN. (1995). Interphase cytogenetic analysis of erbB2 and TopoIIα co-amplification in invasive breast cancer and polysomy of chromosome 17 in ductal carcinoma *in situ*. *Int. J. Cancer*, **64**, 18-26.

NEDERLOF PM, VAN DER FLIER S, RAAP AK AND TANKE HJ. (1992). Quantification of inter and intra-nuclear variation of fluorescence *in situ* hybridization signals. *Cytometry*, **13**, 831-838.

PEDERSEN-BJERGAARD J, JOHANSSON B AND PHILIP P. (1994). Translocation (3;21)(q26;q22) in therapy-related myelodysplasia following drugs targeting DNA topoisomerase II combined with alkylating agents, and in myeloproliferative disorders undergoing spontaneous leukemic transformation. *Cancer Genet. Cytogenet.*, **76,** 50-55.

POMMIER Y, LETEURTRE F, FESEN MR, FUJIMORI A, BERTRAND R, SOLARY E, KOHLHAGEN G AND KOHN KW. (1994). Cellular determinants of sensitivity and resistance to DNA topoisomerase inhibitors. *Cancer Invest.*, **12,** 530-542.

RITKE MK AND YALOWICH JC. (1993). Altered gene expression in human leukemia K562 cells selected for resistance to etoposide. *Biochem. Pharmacol.*, **46,** 2007-2020.

SAMBROOK J, FRITSCH EF AND MANIATIS T. (1989). *Molecular Cloning, A Laboratory Manual.* Second ed. Cold Spring Harbor Laboratory Press: Cold Spring Harbor, New York.

TAN KB, MATTERN MR, ENG WK, MCCABE FL AND JOHNSON RK. (1989). Nonproductive rearrangement of DNA topoisomerase I and II genes: correlation with resistance to topoisomerase inhibitors. *J. Natl Cancer Inst.*, **81,** 1732-1735.

TAN KB, DORMAN TE, FALLS KM, CHUNG TDY, MIRABELLI CK, CROOKE ST AND MAO J. (1992). Topoisomerase-IIα and topoisomerase-IIβ genes-characterization and mapping to human chromosome-17 and chromosome-3, respectively. *Cancer Res.*, **52,** 231-234.

TSUJI T, MIMURA Y, MAEDA K, IDA M, SASAKI K AND SHINOZAKI F. (1994). Numerical aberrations of chromosome 17 detected by FISH with DNA-specific probe in oral tumors. *Anticancer Res.*, **14,** 1689-1694.

VERSANTVOORT CHM, WITHOFF S, BROXTERMAN HJ, KUIPER CM, SCHEPER RJ, MULDER NH AND DE VRIES EGE. (1995). Resistance associated factors in human small cell lung carcinoma $GLC_4$ sublines with increasing adriamycin resistance. *Int. J. Cancer*, **61,** 375-380.

WITHOFF S, SMIT EF, MEERSMA GJ, VAN DEN BERG A, TIMMER-BOSSCHA H, KOK K, POSTMUS PE, MULDER NH, DE VRIES EGE AND BUYS CHCM. (1994). Quantitation of DNA topoisomerase IIα messenger ribonucleic acid levels in a small cell lung cancer cell line and two drug resistant sublines using a polymerase chain reaction-aided transcript titration assay. *Lab. Invest.*, **71,** 61-66.

WOESSNER RD, CHUNG TDY, HOFMANN GA, MATTERN MR, MIRABELLI CK, DRAKE FH AND JOHNSON RK. (1990). Differences between normal and ras-transformed NIH-3T3 cells in expression of 170 kD and 180 kD forms of topoisomerase II. *Cancer Res.*, **50,** 2901-2908.

ZIJLSTRA JG, DE VRIES EGE AND MULDER NH. (1987). Multifactorial drug resistance in an adriamycin-resistant human small cell lung carcinoma cell line. *Cancer Res.*, **47,** 1780-1784.

ZINI N, SANTI S, OGNIBENE A, BAVELLONI A, NERI LM, VALMORI A, MARIANI E, NEGRI C, ASTALDI-RICOTTI GC AND MARALDI NM. (1994). Discrete localization of different DNA topoisomerases in HeLa and K562 cell nuclei and subnuclear fractions. *Exp. Cell Res.*, **210,** 336-348.